# Policy implications of WHO’s Global traditional medicine strategy 2025–2034

**DOI:** 10.2471/BLT.25.293414

**Published:** 2025-09-16

**Authors:** Yuk Ming Alice Wong, Sangyoung Ahn, Aditi Bana, Pradeep Kumar Dua, Rudi Eggers, Shyama Kuruvilla, Yachan Li, Qin Liu, Yunhui Shen, Sungchol Kim

**Affiliations:** a WHO Global Traditional Medicine Centre, World Health Organization, Avenue Appia 20, 1211 Geneva, Switzerland.; b Integrated Health Services Department, World Health Organization, Geneva, Switzerland.

## Abstract

Traditional medicine is used across the world and is deeply rooted in culture, history and local practices. The World Health Organization (WHO)* Global traditional medicine strategy 2025–2034* aims to enhance the contribution of traditional, complementary and integrative medicine to achieving universal health coverage by strengthening the integration of evidence-based traditional, complementary and integrative medicine into national health systems. The strategy prioritizes research to build robust evidence; establishes regulatory mechanisms to ensure safety and quality; promotes integration into health-care services; and fosters cross-sectoral collaboration to utilize traditional, complementary and integrative medicine’s broader health benefits. Despite progress in integrating traditional medicine, challenges persist. For example, lack of robust research methods suited to traditional, complementary and integrative medicine hinders the generation of evidence; variations in regulatory approaches affect safety and quality; and the misappropriation of traditional knowledge raises concerns over the protection of the rights of Indigenous Peoples. Additionally, the lack of standardized models for integration of traditional, complementary and integrative medicine into health systems is an impediment. Drawing on the experience of WHO’s previous strategy on traditional medicine and responding to increased international engagement, the new strategy addresses these challenges and aligns with wider global initiatives on culture, innovation, intellectual property and health governance. The strategy fosters coherence across multilateral agendas and provides a clear path to maximize the contribution of traditional, complementary and integrative medicine while ensuring its safety, quality and accessibility within health-care systems.

## Résumé

La médecine traditionnelle est appliquée dans le monde entier et est profondément ancrée dans la culture, l’histoire et les pratiques locales. La stratégie mondiale de l’Organisation mondiale de la Santé (OMS) pour la médecine traditionnelle 2025–2034 vise à renforcer la contribution de la médecine traditionnelle, complémentaire et intégrative à la réalisation de la couverture sanitaire universelle en renforçant l’intégration de la médecine traditionnelle, complémentaire et intégrative fondée sur des données probantes dans les systèmes de santé nationaux. Cette stratégie accorde la priorité à la recherche afin d’établir des données probantes solides, met en place des mécanismes réglementaires pour garantir la sécurité et la qualité, encourage l’intégration dans les services de santé et favorise la collaboration intersectorielle afin de tirer parti des avantages plus larges de la médecine traditionnelle, complémentaire et intégrative pour la santé. Malgré des progrès réalisés dans l’intégration de la médecine traditionnelle, des défis persistent. Par exemple, l’absence de méthodes de recherche solides adaptées à la médecine traditionnelle, complémentaire et intégrative entrave la production de données probantes; les variations dans les approches réglementaires affectent la sécurité et la qualité; et le détournement des savoirs traditionnels soulève des inquiétudes quant à la protection des droits des peuples autochtones. En outre, l’absence de modèles standardisés pour l’intégration de la médecine traditionnelle, complémentaire et intégrative dans les systèmes de santé constitue un obstacle. S’appuyant sur l’expérience acquise dans le cadre de la précédente stratégie de l’OMS en matière de médecine traditionnelle et faisant suite à un engagement international accru, cette nouvelle stratégie apporte une réponse à ces défis et s’aligne sur des initiatives mondiales plus larges dans les domaines de la culture, de l’innovation, de la propriété intellectuelle et de la gouvernance en matière de santé. Elle favorise la cohérence entre les programmes multilatéraux et trace une voie claire pour maximiser la contribution de la médecine traditionnelle, complémentaire et intégrative, tout en garantissant sa sécurité, sa qualité et son accessibilité au sein des systèmes de santé.

## Resumen

La medicina tradicional se utiliza en todo el mundo y está profundamente arraigada en la cultura, la historia y las prácticas locales. La estrategia mundial de la Organización Mundial de la Salud (OMS) sobre medicina tradicional 2025-2034 tiene como objetivo reforzar la contribución de la medicina tradicional, complementaria e integrativa al logro de la cobertura sanitaria universal mediante la integración de la medicina tradicional, complementaria e integrativa basada en la evidencia en los sistemas nacionales de salud. La estrategia da prioridad a la investigación para generar pruebas sólidas; establece mecanismos regulatorios que garanticen la seguridad y la calidad; promueve la integración en los servicios de atención de salud; y fomenta la colaboración intersectorial para aprovechar los beneficios más amplios de la medicina tradicional, complementaria e integrativa. A pesar de los avances en la integración de la medicina tradicional, persisten desafíos. Por ejemplo, la falta de métodos de investigación sólidos adaptados a la medicina tradicional, complementaria e integrativa dificulta la generación de pruebas; las variaciones en los enfoques regulatorios afectan a la seguridad y la calidad; y la apropiación indebida de los conocimientos tradicionales suscita preocupaciones sobre la protección de los derechos de los pueblos indígenas. Además, la falta de modelos estandarizados para la integración de la medicina tradicional, complementaria e integrativa en los sistemas de salud constituye un obstáculo. Basándose en la experiencia de la estrategia anterior de la OMS sobre medicina tradicional y en respuesta a una mayor participación internacional, la nueva estrategia aborda estos desafíos y se alinea con iniciativas mundiales más amplias en materia de cultura, innovación, propiedad intelectual y gobernanza de la salud. La estrategia fomenta la coherencia entre las agendas multilaterales y proporciona una vía clara para maximizar la contribución de la medicina tradicional, complementaria e integrativa, garantizando al mismo tiempo su seguridad, calidad y accesibilidad en los sistemas de atención de salud.

## ملخص

يُستخدم الطب التقليدي عبر أنحاء العالم، وهو راسخ بعمق في الثقافة، والتاريخ، والممارسات المحلية. وتهدف استراتيجية الطب التقليدي العالمية للفترة من 2025 إلى 2034، والتابعة لمنظمة الصحة العالمية (WHO)، إلى تعزيز إسهام الطب التقليدي والتكميلي والتكاملي في تحقيق التغطية الصحية الشاملة من خلال تعزيز دمج الطب التقليدي والتكميلي والتكاملي القائم على الأدلة، في النظم الصحية الوطنية. وتمنح الاستراتيجية الأولوية لأبحاث بناء أدلة قوية؛ وتضع آليات تنظيمية لضمان السلامة والجودة؛ وتعزز الدمج في خدمات الرعاية الصحية؛ وتدعم التعاون بين القطاعات للاستفادة من المزايا الصحية الأوسع نطاقًا للطب التقليدي والتكميلي والتكاملي. وعلى الرغم من التقدم المحرز في دمج الطب التقليدي، إلا أنه لا تزال هناك تحديات قائمة. فعلى سبيل المثال، يؤدي النقص في أساليب البحث الفعّالة المناسبة للطب التقليدي والتكميلي والتكاملي إلى إعاقة توليد الأدلة؛ كما تؤثر الاختلافات في الأساليب التنظيمية على السلامة والجودة؛ ويؤدي سوء استخدام المعارف التقليدية لإثارة المخاوف بشأن حماية حقوق السكان الأصليين. وبالإضافةً إلى ذلك، فإن غياب نماذج قياسية موحدة لدمج الطب التقليدي والتكميلي والتكاملي في النظم الصحية يُشكّل عائقًا. واعتمادًا على خبرة الناتجة عن استراتيجية منظمة الصحة العالمية السابقة للطب التقليدي، واستجابةً للمشاركة الدولية المتزايدة، تقوم الاستراتيجية الجديدة بالتعامل مع هذه التحديات وتتسق مع المبادرات العالمية الأوسع نطاقًا في مجالات الثقافة، والابتكار، والملكية الفكرية، والحوكمة الصحية. وتُعزز الاستراتيجية التنسيق بين جداول الأعمال متعددة الأطراف، وتُوفر مسارًا واضحًا لتعظيم إسهام الطب التقليدي والتكميلي والتكاملي، مع ضمان سلامته وجودته وإمكانية الحصول عليه في إطار نظم الرعاية الصحية.

## 摘要

传统医学在全世界范围内得以广泛使用，并深深植根于文化、历史和当地实践中。世卫组织 (WHO) 2025–2034 年全球传统医学战略旨在通过推进循证传统、补充和整合医学与国家卫生系统的整合过程，来促使传统、补充和整合医学在实现全民健康覆盖方面发挥更大的作用。该战略优先考虑开展相关研究以收集有力证据，建立监管机制以确保安全性和质量，推动与卫生保健服务的整合过程，并促进跨部门合作以充分利用传统、补充和整合医学带来的更广泛的健康益处。尽管在整合传统医学方面取得了一定进展，但仍面临众多挑战。例如，稳健可靠且适用于传统、补充和整合医学的研究方法的缺乏阻碍了证据的生成，监管方式之间的差异影响了安全性和质量，且传统知识的盗用引发了对土著人民权利能否得到保护的担忧。此外，缺乏将传统、补充和整合医学整合到国家卫生系统的标准化模式，这也是一大障碍。通过借鉴世卫组织以往制定传统医学相关战略的经验并响应日益增长的国际参与需求，该项新战略能够应对上述挑战，并与更广泛的全球性文化、创新、知识产权和卫生治理相关倡议保持一致。该战略旨在促进各类多边议程之间的一致性，并指出了明确的发展方向以最大限度地发挥传统、补充和整合医学的作用，同时确保其在卫生保健系统内的安全性、质量和可及性。

## Резюме

Традиционная медицина используется во всем мире и уходит корнями глубоко в культуру, историю и местные практики. Всемирная организация здравоохранения (ВОЗ) разработала глобальную стратегию в области традиционной медицины на 2025–2034 гг. Цель этой стратегии – усилить роль традиционной, комплементарной и интегративной медицины в достижении всеобщего охвата услугами здравоохранения за счет большего внедрения доказательных практик традиционной, комплементарной и интегративной медицины в национальные системы здравоохранения. Стратегия уделяет первоочередное внимание изысканиям с целью получения надежной доказательной базы, устанавливает регуляторные механизмы для обеспечения безопасности и качества, способствует интеграции в системы первичной медицинской помощи и содействует сотрудничеству различных секторов здравоохранения с целью использования более широких преимуществ для здоровья, которые могут предоставить традиционная, комплементарная и интегративная медицина. Несмотря на прогресс в деле интеграции традиционной медицины, проблемы сохраняются. Например, нехватка надежных методов исследования, учитывающих особенности традиционной, комплементарной и интегративной медицины, затрудняет формирование доказательной базы, различия в регуляторных подходах влияют на безопасность и качество, а неправомерное использование традиционных знаний вызывает обеспокоенность по поводу недостаточной защиты прав коренного населения. Кроме того, препятствием на пути является отсутствие стандартизованных моделей интеграции традиционной, комплементарной и интегративной медицины в системы здравоохранения. Опираясь на опыт предыдущей стратегии ВОЗ в области традиционной медицины и реагируя на усиление международного взаимодействия, новая стратегия направлена на решение этих проблем, и в этом отношении она находится в русле более широких глобальных инициатив в части культуры, инноваций, интеллектуальной собственности и управления в сфере здравоохранения. Стратегия содействует единой направленности многосторонних вопросов, стоящих на повестке дня, и обеспечивает четкий путь максимального использования вклада традиционной, комплементарной и интегративной медицины, гарантируя при этом ее безопасность, качество и доступность в системах здравоохранения.

## Introduction

Traditional medicine is used in all six regions of the World Health Organization (WHO).^1^ Traditional medicine encompasses both codified and non-codified systems,^2^ is deeply rooted in local knowledge, culture and history, and serves as an important health resource, especially in the prevention and management of lifestyle-related chronic diseases and in meeting the health needs of ageing populations.^1^ The 1978 Declaration of Alma-Ata^3^ formally recognized the important role of traditional medicine in primary health care, while the 2018 Declaration of Astana^4^ reaffirmed the importance of traditional knowledge and traditional medicine in achieving universal health coverage (UHC). The use of traditional and complementary medicine is also supported by United Nations General Assembly Resolutions 74/2 (2019)^5^ and 78/4 (2023),^6^ which advocate for the inclusion of safe, evidence-based traditional and complementary medicine in the health-care system to support UHC.

Against this backdrop, global demand for traditional medicine is projected to increase in value from 213.81 billion United States dollars (US$) in 2025 to US$ 359.37 billion by 2032 (compound annual growth rate of 7.7%).^7^ This rising demand highlights the importance of ensuring that traditional medicine is effectively governed and aligned with public health priorities. Responding to these evolving trends and country needs, the WHO *Global traditional medicine strategy 2025–2034*^8^ expands the scope of the existing strategy and clarifies terminology. As part of this refinement, the definitions of traditional medicine and complementary medicine have been updated to better reflect their evolving use and context across countries. At the same time, the definition of integrative medicine has been introduced to acknowledge emerging interdisciplinary approaches and to provide conceptual clarity for developing policies and fostering integration. Integrative medicine is defined as an interdisciplinary and evidence-based approach to health and well-being that combines biomedical and traditional and/or complementary medical knowledge, skills and practices. Together, these definitions underpin the new strategy’s expanded vision that includes traditional, complementary and integrative medicine, and brings together these three approaches to address individual health needs and contribute to the achievement of UHC.

This inclusive approach has been accompanied by steady, measurable progress in traditional, complementary and integrative medicine regulation, research and education, among others, in many WHO Member States, as reflected in the findings of the third WHO global survey on traditional, complementary and integrative medicine (WHO, unpublished report, 2025). In 2023, 90 of the 106 responding Member States reported having national policies on traditional, complementary and integrative medicine, a notable rise from 25 in 1999. National regulations for herbal medicines also expanded, with 116 Member States reporting the implementation of frameworks, up from 65 in 1999. Additionally, 100 Member States reported having national offices dedicated to traditional, complementary and integrative medicine, compared with 49 in 1999, reflecting stronger institutional support. Professional training has also grown, with 58 Member States reporting offering university-level courses on traditional, complementary and integrative medicine, up from 41 in 2012 (WHO, unpublished report, 2025). These achievements, supported by the WHO traditional medicine strategy 2014–2023,^9^ have strengthened national systems and provided a solid foundation for integrating traditional, complementary and integrative medicine into health systems.

Yet challenges remain. The WHO global survey on traditional, complementary and integrative medicine revealed persistent gaps in research capacity: 95% (101/106) of responding WHO Member States cited a lack of research data as a key barrier to the development of traditional, complementary and integrative medicine (WHO, unpublished report, 2025). This finding points to systemic limitations in generating the evidence needed to guide effective policy on and integration of traditional medicine. Additionally, WHO Member States noted a growing demand for cross-sectoral collaboration and equitable benefit-sharing mechanisms for traditional knowledge (WHO, unpublished report, 2025). These findings highlight the importance of advancing strategic directions to address current gaps.

Within this context, the WHO* Global traditional medicine strategy 2025–2034* builds on the steady progress achieved by WHO Member States, while directly addressing their evolving needs. By providing strategic guidance on policy development, regulation, research, and integration of traditional, complementary and integrative medicine, the strategy seeks to transform these challenges into opportunities for stronger, evidence-based and people-centred health systems.

## Global landscape

The new WHO strategy also reflects and builds on the broader global engagement with traditional medicine. In 2024, the World Intellectual Property Organization (WIPO) adopted a new treaty, the WIPO *Treaty on intellectual property, genetic resources and associated traditional knowledge*,^10^ which recognizes the role of the patent system in supporting the protection of genetic resources and traditional knowledge, and promotes transparency and fair benefit-sharing. The United Nations Educational, Scientific and Cultural Organization (UNESCO) has also contributed to this momentum by recognizing both the living traditions and documentary heritage of traditional medicine. On its list of the intangible cultural heritage of humanity, UNESCO has inscribed Indonesia’s jamu wellness culture (2023),^11^ yoga from India (2016)^12^ and traditional Chinese acupuncture and moxibustion (2010),^13^ reflecting the global appreciation of traditional health practices. In parallel, through the memory of the world register, UNESCO acknowledges foundational medical texts such as the *Four treatises of Tibetan medicine* (2023),^14^ China’s *Huang di nei jing* (2011)^15^ and the Korean *Donguibogam* (2009),^16^ highlighting the historical and scientific value of traditional medical knowledge systems. In 2023, WHO, the International Telecommunication Union and WIPO launched the Global Initiative on AI for Health,^17^ to standardize the use of artificial intelligence in health care. This initiative offers opportunities to strengthen the generation of evidence to support decision-making and service innovation relevant to traditional medicine. At the same time, the World Trade Organization (WTO), in collaboration with WHO and WIPO, continues to emphasize equitable access to health-related technologies, including traditional medicine, through its work on intellectual property, trade and innovation.^2^

## Strategic objectives of the strategy

Building on this global momentum, the new WHO strategy outlines how to maximize the contributions of traditional, complementary and integrative medicine to UHC and has four strategic objectives which are outlined in the following subsections.

### Evidence 

As global demand for traditional, complementary and integrative medicine grows, strengthening evidence to support its use is important for safe and effective integration into health systems. Despite widespread use, comprehensive data to validate safety and effectiveness are limited. Expanding research, supported by capacity-building and digital innovations, is essential. The complexity of traditional, complementary and integrative medicine and the holistic, individualized nature of its practices often make conventional pre-clinical and clinical methods challenging, which complicates regulatory approval. To address this issue, the new WHO strategy promotes adaptive trial designs that reflect the holistic nature of traditional medicine to enhance scientific credibility and alignment with global standards.

Advances in scientific research and digital technologies are helping to address these challenges and strengthen the generation of evidence on traditional, complementary and integrative medicine. Omics technologies (that is, high-throughput approaches that provide a comprehensive understanding of biological systems) such as metabolomics for standardization and quality control^18^ and genomics for personalized medicine,^19^ have already improved research outcomes in traditional medicine. Additionally, digital databases, such as the Traditional Chinese Medicine Integrated Database^20^ and the Herb–Drug Interaction Database,^21^ are facilitating predictive modelling and evidence generation. These tools demonstrate the growing potential of integrating advanced scientific methods into traditional, complementary and integrative medicine research, thus helping to connect traditional practices with modern approaches to health care.

### Regulation

Alongside the generation of evidence, ensuring the safety and quality of traditional, complementary and integrative medicine through appropriate regulatory mechanisms is a priority. Regulatory frameworks are necessary to protect the public from unsafe or substandard traditional, complementary and integrative medicine products and services. Yet, varied standards hinder safety, quality and cross-border acceptance.

To address this problem, the new WHO strategy promotes a risk-based regulatory approach tailored to the specific characteristics of traditional, complementary and integrative medicine products and services. The strategy calls for appropriate quality control and participatory mechanisms suited to local contexts. The strategy also emphasizes applying the highest scientific and regulatory standards, as appropriate to national requirements, to ensure safety, quality and effectiveness in clinical settings.

Digital innovation is playing an increasingly important role in strengthening regulatory systems. For example, deoxyribonucleic acid (DNA) barcoding is being used more often to authenticate medicinal plants.^22^ This barcoding supports the quality control of multicomponent herbal formulations by verifying the botanical identity of each ingredient and detecting adulterants or substitutions.^23^ Digital innovation also enables traceability of raw materials^24^ and reduces risks associated with misidentified herbal components. At the same time, drug interactions require enhanced pharmacovigilance systems and clinical research.^25^ The new WHO strategy emphasizes the need for more robust safety monitoring, surveillance and evidence generation on traditional, complementary and integrative medicine in real-world settings.

Additionally, WHO Member States including China,^26^ India^27^ and the Republic of Korea^28^ have integrated digital tools, such as electronic health records and decision-support systems, into regulatory frameworks to enhance regulatory oversight of traditional medicine. While all three countries use digital infrastructure for regulatory purposes, their approaches differ in governance structure, technological emphasis and integration with broader health systems, thus offering complementary models for strengthening the oversight of traditional, complementary and integrative medicine globally. 

### Integration

Building on efforts to strengthen the evidence on traditional, complementary and integrative medicine and ensure regulatory safeguards, the next strategic step is to promote its integration into health systems. This integration plays an important role in reorienting health services, especially as traditional medicine can complement biomedicine services across all levels of care, with primary health care serving as a key entry point to reach communities and address unmet health needs.^8^

However, no universally accepted models for integration of traditional, complementary and integrative medicine exist in the different health-care systems. Country experiences reflect different policy and implementation approaches, particularly in the design of health financing and service delivery mechanisms. Countries such as China,^29^ India^30^ and the Republic of Korea^31^ have integrated traditional medicine into public insurance systems to varying degrees, offering models of national support. 

These different experiences emphasize the importance of context-specific strategies, adapted to national priorities and capacities. To support countries in navigating these different pathways, the new WHO strategy calls for the incorporation of safe and effective traditional, complementary and integrative medicine services into national and local health frameworks, and for policies to facilitate their integration into health systems.

Digital technologies also offer opportunities to enhance the accessibility and delivery of traditional, complementary and integrative medicine. The new WHO strategy promotes the use of digital platforms to support access to, service delivery of and research in traditional, complementary and integrative medicine. Telemedicine and mobile health applications, such as EthnoMed (Harborview Medical Center, Seattle, United States of America),^32^ play a key role in providing remote consultations and education, thus expanding the reach of traditional, complementary and integrative medicine while maintaining safety and quality.

### Collaboration

In addition to its role in health systems, traditional, complementary and integrative medicine provides considerable value across sectors by contributing to cultural preservation, innovation and sustainable development. By engaging with communities and stakeholders, the benefits of traditional, complementary and integrative medicine can extend across multiple sectors and can support sustainable development and well-being.

Traditional medical knowledge has played an essential role in paving the way for the development of key pharmaceuticals such as artemisinin. However, the challenge of misappropriation persists, as seen in the case of turmeric (*Curcuma longa*) and neem (*Azadirachta indica*), among others.^33^ The new WHO strategy emphasizes the proper documentation of traditional medical knowledge, while also raising awareness of ethical considerations related to these practices and the protection of the rights of Indigenous Peoples. Digital tools, such as the Traditional Knowledge Digital Library,^34^ enhance documentation of traditional medicine, improve accessibility and help prevent unauthorized use. Emerging technologies such as blockchain^35^ offer additional transparency and traceability in benefit-sharing arrangements.

In support of pandemic preparedness and global resilience, the new WHO strategy also aligns with the recently adopted WHO Pandemic Agreement.^36^ This agreement, which embeds the One Health approach in its legal framework, aims to strengthen surveillance, equitable access to medical counter-measures and coordinated global action. By integrating traditional medicine within the scope of the Pandemic Agreement, WHO emphasizes that traditional medicine plays a role in pandemic prevention, preparedness and response.

## Synergy with international efforts

The WHO *Global traditional medicine strategy 2025–2034* is designed to create strategic synergy with ongoing global efforts led by other international organizations working in the areas of traditional knowledge, cultural heritage, innovation and trade. The strategy complements these efforts by offering a public health perspective based on WHO’s mandate to advance UHC.

The strategy aligns with the 2024 WIPO *Treaty on intellectual property, genetic resources and associated traditional knowledge*,^10^ as well as the UNESCO Convention for the Safeguarding of Intangible Cultural Heritage,^37^ which recognizes traditional medicine as part of cultural identity. In addition to listing living health traditions, UNESCO’s memory of the world register acknowledges foundational traditional medical texts and underscores their historical, scientific and cultural significance. Through its emphasis on community engagement and the preservation and transmission of traditional knowledge, the new WHO strategy contributes to safeguarding intangible heritage in ways that also advance public health.

In parallel, the new WHO strategy works in concert with the WTO Agreement on Trade-Related Aspects of Intellectual Property Rights (TRIPS),^38^ particularly through the WTO–WHO–WIPO trilateral cooperation,^2^ which connects trade, intellectual property and innovation policies with health equity and access to traditional medical knowledge. Notably, the new WHO strategy complements the Global Initiative on AI for Health,^17^ creating opportunities to apply responsible artificial intelligence in the research, regulation and service innovation of traditional medicine.

The new WHO strategy adds value by translating these global frameworks into practical, health-focused guidance for WHO Member States. This coordinated, multisectoral approach ensures that traditional, complementary and integrative medicine contributes to advancing global health objectives, while supporting coherence across international agendas.

## Conclusion

Recognizing the 2023 United Nations General Assembly Political Declaration of the high-level meeting on UHC, which calls for exploring ways to integrate safe and evidence-based traditional and complementary medicine services within national and local health systems, particularly at the primary health care level in accordance with national contexts and priorities, and acknowledging that primary health care serves as the foundation for UHC, as emphasized in the Declaration of Alma-Ata, the WHO* Global traditional medicine strategy 2025–2034* encourages WHO Member States to develop national policies, strategies and action plans to strengthen the integration of traditional, complementary and integrative medicine into primary health care.

The strategy sets out a vision to enhance research, strengthen regulations, integrate traditional, complementary and integrative medicine into health systems, and optimize multisectoral collaboration. The strategy supports WHO Member States in working to achieve UHC and the health-related sustainable development goals by ensuring that safe, effective, people-centred traditional, complementary and integrative medicine is accessible to all.
